# DNA-guided genome editing using structure-guided endonucleases

**DOI:** 10.1186/s13059-016-1055-4

**Published:** 2016-09-15

**Authors:** Gaurav K. Varshney, Shawn M. Burgess

**Affiliations:** 1 Functional and Chemical Genomics Program, Oklahoma Medical Research Foundation, Oklahoma City, OK 73104 USA; 2 Translational and Functional Genomics Branch, National Human Genome Research Institute, National Institutes of Health, Bethesda, MD 20892 USA

## Abstract

The search for novel ways to target and alter the genomes of living organisms accelerated rapidly this decade with the discovery of CRISPR/Cas9. Since the initial discovery, efforts to find alternative methods for altering the genome have expanded. A new study presenting an alternative approach has been demonstrated that utilizes flap endonuclease 1 (FEN-1) fused to the Fok1 endonuclease, which shows potential for DNA-guided genome targeting in vivo.

## Introduction

With the explosion of interest in “genome editing” arising following the demonstration that Cas9 acts as an RNA-guided nuclease (i.e., RNA sequences are used to guide nuclease activity to a specific DNA sequence), researchers have worked tirelessly to discover novel ways to manipulate the genome and gene expression. This effort has resulted in a number of new genes and approaches using other RNA-guided nucleases, DNA-guided nucleases, synthetic transcription factors, and other exciting techniques. The latest approach, published in the current issue of *Genome Biology* [1], uses an enzyme involved in DNA repair and replication known as flap endonuclease 1 (FEN-1) fused to the Fok1 endonuclease. Xu and colleagues [1] have shown that this strategy results in a DNA-guided nuclease that, when injected, can efficiently cause large deletions in the zebrafish genome in vivo. This represents a significant new tool in the genome editing toolbox.

## RNA-guided genome editing

Targeted genome engineering has come a long way since the first publication describing the zinc-finger fusions to Fok1 endonuclease in 1996. For many years, programmable zinc-finger nucleases (ZFNs), and more recently transcription activator-like effector nucleases (TALENs), were used for generating targeted genomic manipulations [2]. Each target of these nucleases involved a re-engineering of the protein directly, so these approaches required significant levels of expertise and were often laborious to construct. Thus, they were not widely adopted. In August 2012, an international team of researchers led by Jennifer Doudna and Emmanuelle Charpentier published a landmark paper describing the use of the class II CRISPR/Cas9 system from *Streptococcus pyogenes* for gene editing. They demonstrated that three of the components (crRNA, tracrRNA, and Cas9 protein) could be used to generate DNA double-stranded breaks in vitro in a sequence-specific manner [3]. The simplicity and robustness of this approach has resulted in no less than a revolution in genome editing in less than a decade [4].

For CRISPR/Cas9, any sequence in the genome can be targeted that has a protospacer adjacent motif (PAM) immediately downstream of the target site (NGG or NAG for spCas9) and this targeting has worked in essentially every organism tested [4]. Cas9 proteins from different bacterial species have different PAM sequences and many of them are being tested for their utility as genome-editing tools [5]. Thus far, CRISPR/Cas9-based tools are being used for a constantly growing list of applications, including genomic modifications, epigenetic regulation, functional-genomics screens, live imaging of genomes, and gene therapy [4, 5]. The quest for expanding the CRISPR-based genome editing toolbox has uncovered many other similar proteins by analyzing microbial genomes and metagenomic data. In such studies, researchers have discovered other members of class II CRISPR systems, such as Cpf1, C2c1, C2c2, and C2c3 [6]. The endonuclease Cpf1 has been shown to work in in vivo genome editing; C2c2 has endoribonuclease activity with the ability to edit RNA; and these other enzymes could further revolutionize the genome editing toolbox. By increasing the number of available PAM sites with new class II components such as Cpf1, or targeting RNA instead of DNA in the case of C2c2, the “target space” of the genome increases, making more types of editing possible.

## DNA-guided genome editing

### Genome editing mediated by *Natronobacterium gregoryi* NgAgo

The rapid progress adapting Cas9 into a ubiquitous tool of molecular biology research further motivated researchers to look for additional alternatives for genome editing. In this quest, a group from China led by Chunyu Han has developed a DNA-guided genome editing method using the Argonaute protein from *Natronobacterium gregoryi* (NgAgo) [7]. Argonaute from *Thermo thermophiles* (TtAgo) has previously been shown to edit plasmid DNA at non-physiological temperatures (>65 °C) [8]. In the Han group publication, the Argonaute protein NgAgo was able to edit DNA in cell culture at 37 °C. The NgAgo-mediated genome editing requires a 5′-phosphorylated 24-nucleotide DNA guide and the Argonaute protein. This new method generated tremendous excitement within the scientific community, in part because, in contrast to CRISPR/Cas9, NgAgo did not have any sequence constraints. Han and colleagues showed that purified Argonaute protein, together with a guide DNA, could cleave plasmids in vitro. While these results were very exciting, reproducibility has been a nagging issue and the utility or validity of this approach is still in question [9].

### Genome editing mediated by a structure-guided endonuclease

A recent study published in the current issue of *Genome Biology* by Xu and colleagues potentially adds yet another tool—structure-guided endonuclease (SGN)—to the rapidly increasing genome-editing toolbox [1]. Three key features of this approach are that, first, the FEN-1 fusion can use DNA oligomers to target a specific locus; second, targeting using this approach has a tendency to create larger deletions on the order of several hundreds to thousands of bases, and finally, the authors were able to demonstrate that this approach works in zebrafish embryos, showing that targeting is possible in an animal model.

**Fig. 1:**
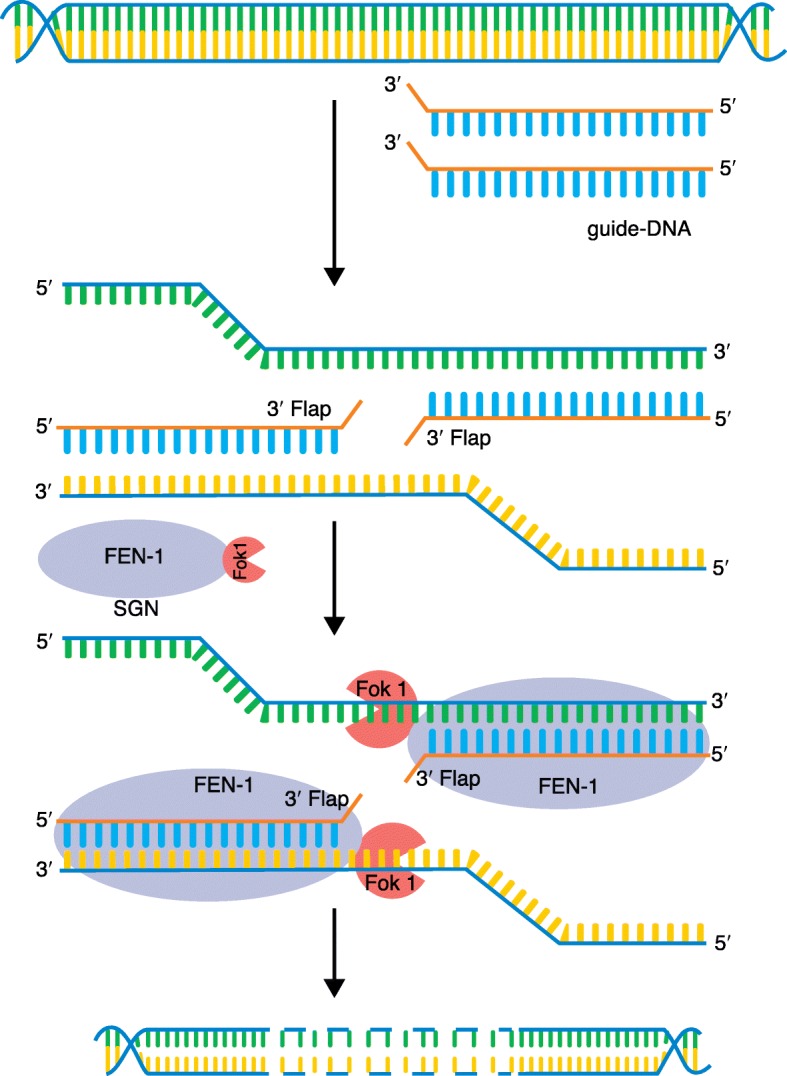
Genome editing using a structure-guided endonuclease (SGN). SGN-mediated genome editing has two components: a SGN consisting of the FEN-1 enzyme fused with the Fok1 endonuclease and two 20–60-nucleotide target sequences with single, 3′ unpaired bases (*3′ Flap*). The two guides bind to the complementary sequences and the FEN-1 component of SGN recognizes the 3′ flap structure and guides the Fok1 dimer into position to generate a double-stranded cut, which then repairs by non-homologous end joining after what appears to be an expansion of the deleted region by a currently unknown mechanism

Structure-guided nuclease-mediated DNA editing uses an engineered SGN comprising FEN-1, which recognizes a 3′ “flap” structure (consisting of a double-stranded helix where one strand is shorter, creating a flap at the end; Fig. 1), and the cleavage domain of the Fok1 endonuclease. FEN-1 uses a guide DNA comprising a (minimum) 20 base-pair (bp) complementary sequence to the target site where the 3′ end has a single-base mismatch creating an unpaired base, forming the “flap” structure. Similar to ZFNs and TALENS, in the SGN strategy, the two halves of the Fok1 endonuclease are brought together by two adjacent targets on opposite strands, in essence creating a 40-bp or longer target sequence (Fig. 1).

Xu and colleagues tested different lengths of guide DNA, ranging from 10–60 nucleotides. SGN failed to cleave targets that were less than 20 nucleotides but good cutting efficiency was achieved for 20–60 bp DNA guides. Although the authors were able to demonstrate convincingly that SGN cuts single-stranded target DNA using guide DNAs of 20–60 nucleotides, no quantification of cutting efficiencies was reported. Based on visual inspection of the polyacrylamide gel electrophoresis (PAGE) results, it is possible that the guide DNAs of length 50 nucleotides were the most efficient size for cutting, but additional experiments are needed to verify this possibility and to evaluate the general efficacy of SGNs in vivo. Given that a 3′ unpaired flap is essential for FEN-1 to recognize the target, Xu and colleagues tested all possible 3′ nucleotide mismatches (C-T, G-T, T-T, C-A, G-A, A-A, C-C, A-C, T-C, A-G, T-G, G-G) and were not able to detect any differences in their DNA cutting efficiencies. More quantification data will be helpful in determining the effect of unpaired bases on the cutting efficiency.

SGNs cut the target sites 9–10 nucleotides away from the 3′ end of the guide DNA. Xu et al. extended their in vitro studies by testing the ability of SGNs to edit genes in vivo by using zebrafish embryos. First, they injected two guide DNAs targeting enhanced green fluorescent protein (eGFP) with SGN mRNA into one-cell-stage embryos. These injections generated mutations in up to 25 % of the sequenced eGFP DNA. The authors also tested the effect of distance between the two guides on mutagenesis efficiencies. The guide DNA pairs spaced by 0, 8, 18, 32, and 50 bp generated mutations with 4, 0, 6.5, 18, and 25 % efficiencies, respectively. In addition to eGFP, the authors targeted two zebrafish genes—*znf703* and *cyp26b1*—and were able to generate genomic mutations with ~10 % efficiency. The mutagenic efficiencies were low compared with those of CRISPR-Cas9 but there are still significant opportunities to optimize the efficacy of the approach.

For ZFNs, TALENs, and CRISPR/Cas9, one common feature is that the insertions and deletions generated by the double-stranded break are typically small, most being on the order of just a few nucleotides to tens of nucleotides. The in vivo deletions identified using an SGN approach were much larger, ranging from approximately 650 to 2600 bp. It is currently unknown why these larger deletions are being generated but it potentially has something to do with the normal functions of FEN-1, which has been implicated in both DNA repair and replication. The combination of Fok1 activity and FEN-1 together might cause a “chain reaction” that creates deletions larger than those simple double-stranded breaks generated by the other techniques. When targeting genes for inactivation, it is obviously advantageous to be able to generate larger deletions to ensure that gene function is truly disrupted. Occasionally false-negatives can arise because a smaller, frame-shifting mutation is masked biologically by compensation mechanisms. A larger deletion can prevent these potential errors.

## Concluding remarks

In the rapidly changing landscape of genome editing, the SGN approach is an exciting new option. The flexibility and simplicity of DNA-guided genome targeting is a major strength, as is its potential for generating larger deletions. Given the endogenous DNA repair functions of FEN-1, it will be interesting to see in the future whether there is a potential to stimulate specific changes in sequence using repair templates. With validation from other laboratories, the work of Xu and colleagues could result in an important alternative to RNA-guided Cas9 for genome engineering.

## Abbreviations

eGFP: Enhanced green-fluorescent protein
FEN-1: Flap endonuclease 1
NgAgo: *Natronobacterium* gregoryi Argonaute protein
PAM: Protospacer adjacent motif
SGN: Structure-guided endonuclease
TALEN: Transcription activator-like effector nuclease
TtAgo: *Thermo thermophiles* Argonaute protein
ZFN: Zinc-finger nuclease

## References

[1] Xu S, Cao S, Zou B, Yue Y, Gu C, Chen X, et al. An alternative novel tool for DNA editing without target sequence limitation: the structure-guided nuclease (SGN). Genome Biol. 2016. doi:10.1186/s13059-016-1038-5. 10.1186/s13059-016-1038-5 PMC5025552 27634179

[2] Kim H, Kim JS (2014). A guide to genome engineering with programmable nucleases. Nat Rev Genet.

[3] Jinek M, Chylinski K, Fonfara I, Hauer M, Doudna JA, Charpentier E (2012). A programmable dual-RNA-guided DNA endonuclease in adaptive bacterial immunity. Science.

[4] Sander JD, Joung JK (2014). CRISPR-Cas systems for editing, regulating and targeting genomes. Nat Biotechnol.

[5] Hsu PD, Lander ES, Zhang F (2014). Development and applications of CRISPR-Cas9 for genome engineering. Cell.

[6] Mohanraju P, Makarova KS, Zetsche B, Zhang F, Koonin EV, van der Oost J (2016). Diverse evolutionary roots and mechanistic variations of the CRISPR-Cas systems. Science.

[7] Gao F, Shen XZ, Jiang F, Wu Y, Han C (2016). DNA-guided genome editing using the *Natronobacterium gregoryi* Argonaute. Nat Biotechnol.

[8] Swarts DC, Jore MM, Westra ER, Zhu Y, Janssen JH, Snijders AP (2014). DNA-guided DNA interference by a prokaryotic Argonaute. Nature.

[9] Cyranoski D (2016). Replications, ridicule and a recluse: the controversy over NgAgo gene-editing intensifies. Nature.

